# Critical dysregulated signaling pathways in drug resistance: highlighting the repositioning of mebendazole for cancer therapy

**DOI:** 10.3389/fphar.2025.1631419

**Published:** 2025-07-25

**Authors:** Amin Aliabadi, Seyed Zachariah Moradi, Sadaf Abdian, Sajad Fakhri, Javier Echeverría

**Affiliations:** ^1^ Department of Pharmacology, School of Medicine, Shiraz University of Medical Sciences, Shiraz, Iran; ^2^ Student Research Committee, School of Medicine, Shiraz University of Medical Sciences, Shiraz, Iran; ^3^ Pharmaceutical Sciences Research Center, Health Institute, Kermanshah University of Medical Sciences, Kermanshah, Iran; ^4^ Student Research Committee, Kermanshah University of Medical Sciences, Kermanshah, Iran; ^5^ Departamento de Ciencias del Ambiente, Facultad de Química y Biología, Universidad de Santiago de Chile, Santiago, Chile

**Keywords:** mebendazole, cancer drug resistance, drug repositioning, angiogenesis, apoptosis, signaling pathways

## Abstract

**Background:**

Cancer drug resistance significantly reduces the effectiveness of current anticancer treatments. Multiple dysregulated signaling pathways drive cancer initiation, progression, and related drug resistance. This highlights the need for developing new multi-targeting drugs that are more cost-effective, have fewer side effects, and remain effective against cancer. Drug repurposing offers a promising solution to expensive targeted therapies and helps overcome drug resistance. Mebendazole (MBZ), albendazole, flubendazole, and oxfendazole are broad-spectrum anti-helminthic drugs from the benzimidazole family.

**Purpose:**

Therefore, MBZ demonstrated potential in suppressing the growth of various cancer cells, both *in vitro* and *in vivo*. Consequently, we thoroughly reviewed MBZ as a therapeutic option against cancer and related drug resistance.

**Results and discussion:**

In this study, we identified MBZ as a promising cancer treatment that works through multiple mechanisms such as regulating tumor angiogenesis, autophagy, and apoptosis, modulating key signaling pathways, boosting antitumor immune responses, and inhibiting matrix metalloproteinases activity—all of which are major factors in cancer drug resistance. Additionally, the development of new MBZ delivery systems aims to address its pharmacokinetic limitations. While the anticancer effects of MBZ are encouraging, further research is needed before it can be used clinically.

**Conclusion:**

Extensive data from *in vitro*, *in vivo*, and clinical trials support MBZ’s anticancer potential and highlight the need for innovative delivery methods, including polymeric nanoparticles, nanostructured lipid formulations, micelles, nanosuspensions, and beyond.

## 1 Introduction

Cancer is the uncontrolled development of unusual cells within the body. Although cancer has long been identified, recent advances paved the way for the treatment of cancer (Fakhri et al., 2022b). The cancer treatment process is mainly based on developing methods for elective surgery, radiation therapy, chemotherapy drugs, and targeted therapies (Bold et al., 1997; Sawyers, 2004). Drug resistance and cancer cell survival are critical issues in the progression of cancer. So, overcoming this problem is one of the major challenges in treating various cancers (Baghban et al., 2023). This line reveals the detailed molecular mechanisms of cancer cell survival, paving the way for the discovery of novel therapeutic approaches in cancer treatment (Jacks and Weinberg, 2002). Several molecular mechanisms, including microtubule disruption, angiogenesis, apoptosis, autophagy, immune response, Hedgehog signaling, matrix metalloproteinase (MMP), and other involved signaling pathways, are essential for the survival of cancer cells (Hein et al., 2014; Mutschelknaus et al., 2016; Das et al., 2018).

As a strategy for identifying novel therapeutic agents, drug repositioning (finding new therapeutic uses for existing or old drugs) has proven effective in discovering or developing new therapeutic options (Pushpakom et al., 2019). Therefore, offering a new approach with low toxicity, pre-clinical efficacy, and pharmacokinetics that allow therapeutic concentrations at the tumor site, synergy with approved anticancer therapies, affordability, and the potential to combat cancer drug resistance makes it an ideal candidate for drug repurposing in cancer (Oprea and Mestres, 2012; Sleire et al., 2017).

Benzimidazoles are heterocyclic and aromatic organic compounds. They are bicyclic compounds formed by the fusion of benzene and imidazole ring systems, which are essential pharmacophores and privileged structures in medicinal chemistry. Variations in the position of the benzimidazole ring (such as the phenyl ring at positions 1 and 2) can be used to develop target-specific derivatives. Mebendazole (MBZ, benzoyl + carbamate), albendazole (thioether + carbamate), tiabendazole (thiazole ring), flubendazole, and oxfendazole (phenylthio/sulfoxide chain) are broad-spectrum anti-helminthic drugs in the benzimidazole family (Shrivastava et al., 2017; Satija et al., 2022). Generally, benzimidazoles stop parasitic cell growth by binding to tubulin subunits in the parasite’s intestinal epithelium, which prevents tubulin polymerization (Pantziarka et al., 2014; Pinto et al., 2015). Among the benzimidazole family, MBZ is widely used worldwide to treat helminthic diseases in humans. Additionally, several studies have demonstrated that MBZ has a significant impact on the growth of various cancer cells (Bai et al., 2011).

MBZ employs several mechanisms to target tumor cells in cancer treatment. It inhibits the growth and proliferation of cancerous cells by preventing microtubule formation (Mukhopadhyay et al., 2002; De Witt et al., 2017). MBZ also suppresses tumor angiogenesis and stimulates autophagy in cancer cells (Bai et al., 2015; Son et al., 2020). Additionally, MBZ triggers apoptosis in various cancer cell lines by balancing apoptotic mediators such as B-cell leukemia/lymphoma 2 (Bcl-2)/Bcl-2-associated X protein (Bax), tumor protein p53 (p53), and X-linked apoptosis inhibitor (XIAP) (Son et al., 2020; Chai et al., 2021). It also inhibits metastasis, reduces the formation and size of primary tumors, and prevents liver and lung metastases (Choi et al., 2021; Joe et al., 2022). MBZ induces the production of reactive oxygen species and DNA damage in tumor cells (Petersen and Baird, 2021). Moreover, MBZ, when combined with radiotherapy, enhances the toxicity of natural killer cells and decreases cancer cell transformation (Choi et al., 2022). It modulates several signaling pathways critical to cancer progression, including Signal Transducer and Activator of Transcription 1 and 2 (STAT1/2), E-26-like protein 1 (ELK1)/serum response factor (SRF), Activator protein-1 (AP1), and c-myc proto-oncogene (MYC)/myc-associated factor X (MAX). Additionally, MBZ boosts the efficacy of anticancer drugs such as cisplatin (Zhang et al., 2017) and docetaxel (Rushworth et al., 2020).

In various studies on tumor cells, MBZ has shown multiple mechanisms that exhibit anticancer effects. These include microtubule disruption, inhibition of tumor angiogenesis, regulation of autophagy, suppression of apoptosis, and attenuation of key signaling pathways in cancer cells, as described in the following sections. Several recent reviews have highlighted the signaling pathways involved in cancer drug resistance (Ward et al., 2021; Xiong et al., 2022; Fakhri et al., 2023; Dong et al., 2024; Nunes et al., 2024; Yaylim et al., 2024). Additionally, recent reviews point to the anticancer potential of benzimidazoles (Son et al., 2020; Chai et al., 2021). However, in this review, we focus on the mechanistically based anticancer activity of MBZ. Since MBZ addresses multiple mechanisms of anticancer drug resistance and appears to possess all the properties of an effective anticancer drug (Guerini et al., 2019), we conducted a comprehensive review of its potential as a candidate therapeutic agent for cancer treatment. This is a novel, in-depth review of the mechanistic repositioning of MBZ in cancer drug resistance, highlighting innovative delivery systems.

### 1.1 The anticancer activity of MBZ in combating drug resistance: mechanistic approaches

Multiple mechanisms contribute to cancer drug resistance, including increased expression of adenosine triphosphate (ATP)-binding cassette (ABC) transporter-mediated drug extrusion [e.g., P-glycoprotein (P-gp/ABCB1)], breast cancer resistance protein (BCRP/ABCG2), multidrug resistance protein 1 (MRP1/ABCC1), impaired DNA repair, growth factor receptor (GFR) dysregulation, inhibition of downstream cell death pathways, activation of survival signaling pathways, altered drug metabolism, gene amplification, transfer and expression of miRNAs, pre-existing or acquired tumor cell heterogeneity—especially cancer stem-like cells (CSCs)—fibrotic responses, hypoxia, and immune activation, as well as dysregulation of interconnected inflammation, apoptosis, and oxidative stress pathways (Fakhri et al., 2023). MBZ utilizes several mechanisms of action to counteract cancer initiation, progression, metastasis, and resistance. These include microtubule disruption, induction of apoptosis, inhibition of angiogenesis, induction of autophagy, stimulation of antitumor immune responses, and regulation of multiple signaling pathways.

#### 1.1.1 Microtubule-disrupting activity

Microtubules serve as intracellular scaffolds, and cellular functions largely rely on their unique polymerization dynamics. Additionally, dysfunction of the cytoskeleton causes intracellular stress. Moreover, the disruption in the balance between tubulin monomers and dimers, as well as microtubule polymers, can activate signaling stress (Chai et al., 2021). Microtubule-stabilizing agents, such as paclitaxel and docetaxel, and microtubule-destabilizing agents, including vinblastine, vincristine, nocodazole, and colchicine, are known to interfere with normal microtubule functions, leading to cell cycle arrest at the G2-M phase (Jordan and Wilson, 1998; Zhou and Giannakakou, 2005).

Several microtubule-targeting drugs have demonstrated significant preclinical and clinical activity against tumor cells. However, due to the toxicity of such drugs, their administration has been limited (Zhang et al., 2020). The effects of MBZ on mammalian tubulin were first demonstrated in a series of published reports, which showed that it inhibits mitosis in the cell cycle (Foster et al., 1987; Kamal et al., 2015). Structure-activity relationship-based reports indicated that, unlike other anticancer drugs, even high doses of MBZ are significantly safe in humans when administered in clinical settings (Mudduluru et al., 2016; Huang et al., 2021). In this context, a 10 mg/kg oral administration of MBZ, administered for 5 days to mice, showed no toxicity to the liver and kidneys, and had no adverse effects on animal weight. Additionally, MBZ significantly inhibited the transformation of tumoral cells at low doses (Choi et al., 2021). MBZ demonstrated anticancer effects against vincristine in a glioma mouse model. It also increased the survival of mice (Skibinski et al., 2018), unlike vincristine (De Witt et al., 2017). MBZ mitigated the adverse effects of standard anticancer therapies by adjusting and reducing their doses and enhancing their effectiveness (Son et al., 2020). Additionally, MBZ can exhibit greater sensitivity in tumor cells compared to normal cells and show less toxicity compared to anticancer drugs (Son et al., 2020; Meco et al., 2023). From the mechanistic pathway, MBZ stops the cell cycle at the G2-M interval, which eventually leads to the apoptosis of cancer cells (Mukhopadhyay et al., 2002). Some clinical studies have also been done on the safety of MBZ (NCT01173562).

The binding site of MBZ to tubulin is similar to that of colchicine. A large pocket surrounded by two strands of β-tubulin sheets and two α-helixes (H7 and H8) forms the colchicine-binding site. Also, this region is covered by two loops (βT7 and αT5) (Dorléans et al., 2009). Colchicine targets the β-tubulin subunit of the microtubule and prevents the formation of a straight structure. This may inhibit the assembly of microtubules by MBZ (Ravelli et al., 2004; Dorléans et al., 2009).

Overall, MBZ demonstrated safe microtubule-disrupting activity, even at high doses, making it a potential anticancer agent.

#### 1.1.2 Tumor angiogenesis

Angiogenesis inhibitors that target the vascular endothelial growth factor (VEGF) signaling pathway demonstrate acceptable therapeutic efficacy; however, tumors have been observed to exhibit inherent resistance to anti-angiogenic agents in clinical studies (Pietras et al., 2008). Vascular endothelial growth factor receptor-2 (VEGFR2) is a functional receptor that mediates the majority of VEGF cellular responses. After VEGF binding, VEGFR2 undergoes auto-phosphorylation, thus activating downstream signaling pathways. Recently, clinical studies have demonstrated that MBZ can be an effective treatment for cancer by inhibiting tumor angiogenesis (Bai et al., 2015). Accordingly, some studies showed that MBZ can inhibit the auto-phosphorylation of VEGFR2 (Y1175), which in turn provides a binding site for phospholipase C gamma (PLCγ) and leads to the phosphorylation of extracellular signal-regulated kinase 1/2 (ERK1/2) (Bai et al., 2015). Accordingly, and due to the MBZ’s multi-targeting anticancer effects, its co-administration with anti-angiogenic drugs results in synergistic effects, overcoming resistance, and enhancing immune responses.

#### 1.1.3 Autophagy

Autophagy is a physiological process in which the cell destroys waste materials in the cytoplasm, such as old organelles, bacteria, and viruses, by forming membrane structures called autophagosomes and combining them with lysosomes (Zhang et al., 2020). Autophagosome formation is initiated by class III phosphoinositide 3-kinase (PI3K) and the primary regulator of autophagy initiation, Beclin 1. Additionally, autophagy genes 5 (ATG5) and 7 (ATG7) play a crucial role as major proteins in this process. The primary role of autophagy is to coordinate the cell’s response to cellular stress, preventing apoptosis under certain conditions while inducing cell death in other cellular conditions (Kumar et al., 2022). Some angiogenesis inhibitors promote cell survival by inducing autophagy, while other drugs induce cell death (Kardideh et al., 2019). Flubendazole and MBZ are drugs that have a similar structure and induce autophagy. Magnolol derivatives might repress angiogenesis by activating autophagy and leading to cell death, independent of apoptosis, in human umbilical vein endothelial cells and PC-3 cells (Sung et al., 2019). Studies have shown that autophagy inhibition reverses the anti-angiogenic property of magnolol derivatives (Kumar et al., 2013).

Several studies have demonstrated that anti-angiogenic inhibitors can enhance cell survival by inducing autophagy, which may be effective in combating cancer cell resistance to anti-angiogenic treatments (Belloni et al., 2010). For example, endostatin is an angiogenic inhibitor that causes autophagy by increasing Beclin1 (Nguyen et al., 2009). As a result, it is possible that MBZ could also lead to an autophagy process, repress angiogenesis, and cause cell death independent of apoptosis (Kumar et al., 2013). Other studies have shown that flubendazole, a benzimidazole derivative, is a potent stimulator of autophagy. This compound affects autophagy regulators, including Beclin1, Transcription factor EB (TFEB), mammalian target of rapamycin (mTOR), and α-tubulin *N*-acetyltransferase (ATAT) by inducing microtubule acetylation and disrupting dynamic microtubules (Chauhan et al., 2015). Furthermore, Sung et al. showed that MBZ induces autophagy to protect the endothelial cells (ECs) against cell death and interferes with the autophagy response. The observed effect is an augmentation of the inhibitory activity of MBZ on the behavior of endothelial cells (Sung et al., 2019).

#### 1.1.4 Apoptosis

The paucity of reaction towards a broad spectrum of anticancer treatments suggests the existence of intricate mechanisms underlying drug resistance. Numerous lines of evidence indicate that abnormalities in the signaling cascades that facilitate programmed cell death, also known as apoptotic pathways, may have significant implications for the progression of malignant neoplasms (Kardideh et al., 2019). Reports have shown that the proapoptotic and apoptotic inhibitor mediators [e.g., myeloid cell leukemia 1 (Mcl-1), B-cell lymphoma-extra large (Bcl-XL), and Bcl-2] could be candidate therapies for the treatment of cancers. There is substantial evidence to suggest that MBZ exerts inhibitory effects on cancer cell growth, particularly in the case of melanoma, by triggering an apoptotic response (Son et al., 2020). The intrinsic apoptotic response, mediated by Bcl-2 phosphorylation, occurs rapidly in melanoma cells after treatment with MBZ. Phosphorylation of Bcl-2 in melanoma cells prevents it from interacting with Bax, a pro-apoptotic protein, thereby causing apoptosis. Melanocytes that resist MBZ have the potential to increase sensitivity by reducing Bcl-2 protein levels, indicating their indispensable involvement in the cellular response to MBZ-induced disruption of tubulin (Doudican et al., 2008). MBZ could also lead to p53 accumulation and cell cycle arrest in the G2-M stage, resulting in time- and dose-dependent apoptosis. Additionally, MBZ-mediated apoptosis could involve XIAP. XIAP is a protein that potently prevents apoptosis by inhibiting caspases 3, 7, and 9 (Chai et al., 2021). Studies have also shown that increased expression of XIAP led to the development of melanoma. The investigation conducted on the impact of MBZ on human melanoma cell lines M-14 and A-375 demonstrated that the administration of MBZ resulted in a reduction of XIAP levels over time. Moreover, this reduction exhibited an inverse correlation with escalated rates of apoptosis, caspase 9 activation, and cleaved ADP-ribose polymerase (PARP) markers (Hiscutt et al., 2010; Doudican et al., 2013). The overall antiapoptotic mechanisms of MBZ showed its anticancer potential.

## 2 Regulation of effective signaling pathways in cancer by MBZ

### 2.1 ERK signaling pathway

Mitogen-activated protein kinases (MAPKs) could be classified into three prominent protein families, including the p38 proteins, ERK, and stress-activated protein kinases/c-Jun N-terminal kinase (SAPK/JNK). Furthermore, it should be noted that Ras, Raf, and MAP–ERK kinase (MEK) are proteins with proximal positioning in the ERK pathway (Fan and Chambers, 2001; Kim and Choi, 2010). Studies have demonstrated that ERK plays a crucial role in cell survival, activation, and differentiation. Andersson et al. have shown that MBZ passed immune-modulating activity through the ERK signaling pathway (Andersson et al., 2020). From the mechanistic insight, MBZ makes a specific interaction with the tubulin structure. It also generally binds to the colchicine-binding domain of tubulin; however, such an effect has also been reported with other benzimidazoles. Furthermore, the inhibition of the dual specificity tyrosine-phosphorylation-regulated kinase 1B (DYRK1b) enzyme by marginal concentrations of MBZ may trigger the activation of ERK. Consistently, MBZ strongly inhibits BRAF8 in wild-type RAF cells, leading to inconsistent ERK activation. Additionally, ataxia telangiectasia-mutated (ATM) kinase inhibition effectively decreases the apoptosis induced by MBZ in the zebrafish retina (Gao et al., 2013; Nygren et al., 2013).

### 2.2 Hedgehog signaling pathway

The involvement of Hedgehog (Hh) signaling pathways in several types of cancer renders them attractive targets for novel anticancer agents. The Hh signaling pathway initiates through the interaction between Hh ligands and the patched 1 (PTCH1) receptor. In the absence of a bound ligand, PTCH1 impedes the initiation of Smoothened (SMO), a frizzled class receptor within the primary cilium, which plays a crucial role in the transmission of diverse chemical and mechanical signals. In the presence of a ligand, PTCH1 undergoes dissociation from the cilium. It instigates the activation of downstream effectors, including the transcription factor glioma-associated oncogene (GLI), in a process orchestrated by the activation of SMO. Genetic mutations in the gene encoding PTCH1 or aberrations in the Hh signaling pathways are frequently observed in numerous types of cancer (Yauch et al., 2008; Guerini et al., 2019).

It has been demonstrated through empirical observation that the activation of the Hh signaling pathway is evident in many gliomas, melanomas, lung cancers, ovarian cancers, adrenal cancers, and colon cancers, all of which respond to MBZ. Inhibition of primary cilia formation, attenuation of downstream Hedgehog pathway effectors’ expression, as well as reduction in the proliferation and survival of human medulloblastoma cells, were accomplished through the administration of MBZ. In addition, MBZ inhibited the activation of SMO mutant proteins (Larsen et al., 2015; Jain et al., 2020; Staedtke et al., 2020).

By regulating effective signaling pathways in cancer (e.g., ERK and Hh), MBZ could potentially exhibit anticancer effects.

### 2.3 Inducing antitumor immune responses

Rapid innate immune responses to infection depend on pattern recognition receptors produced by cells of the innate immune system. These receptors identify molecules associated with pathogenic agents. Because they are located in repetitive patterns of pattern recognition receptors on the surface of professional phagocytes such as dendritic cells, macrophages, and neutrophils, they receive pathogens and deliver them to lysosomes for destruction (Murray, 2017). Macrophages are divided into two subtypes, M1 and M2. M1 macrophages exhibit anti-tumor effects due to their phagocytic and antigen-presenting activity and the production of Th-1 activating cytokines. On the other hand, M2 macrophages promote cancer cell proliferation by stimulating matrix remodeling, angiogenesis, and immune tolerance (Aras and Zaidi, 2017). The regulatory function of MBZ has been observed to regulate the transcription of specific cytokine genes - notably tumor necrosis factor (TNF), interleukin 6 (IL-6), and 8 (IL-8) - that are associated with the M1 phenotype, together with the expression of surface markers CD80 and CD86, and T cell chemokines. In contrast, no alterations in the expression of M2 markers were observed. Additionally, exposure to MBZ resulted in the production of interleukin-1 (IL-1), while the administration of other benzimidazoles had no discernible impact on the release of IL-1. In line with this, it can be inferred that MBZ can intensify the immune-stimulatory and anticancer properties of anti-CD3/IL2-activated peripheral blood mononuclear cells (PBMCs), thereby suggesting its potential contributions to the anticancer mechanisms (Rubin et al., 2018).

Additionally, another effect of MBZ in inducing the antitumor response is through the inhibition of DYRK1B, a mediator of immune-modulating activity. Accordingly, DYRK1B inhibition could provide a limited role in the development of MBZ-induced immune reactions. Therefore, the inhibitory effects of MBZ on DYRK1B are implicated as a contributing aspect of the mechanism of action that prompts the polarization of M2 towards M1 macrophages, ultimately eliciting anticancer responses (Blom et al., 2019).

### 2.4 Suppressing matrix metalloproteinase 2 activity

MMPs are the most critical gelatinases and are produced and secreted by several cell lines, including fibroblasts, leukocytes, and tumor cells. The expression of metalloproteinases is influenced by a multitude of factors, including cytokines, growth factors, oncogenes, intercellular interactions, and extracellular matrix components (Mondal et al., 2020). MBZ significantly reduced the activity of MMP-2 in different concentrations, while it was ineffective on the activity of MMP-9 (Pinto et al., 2015).

### 2.5 MBZ combination with radiotherapy against cancer

**FIGURE 1:**
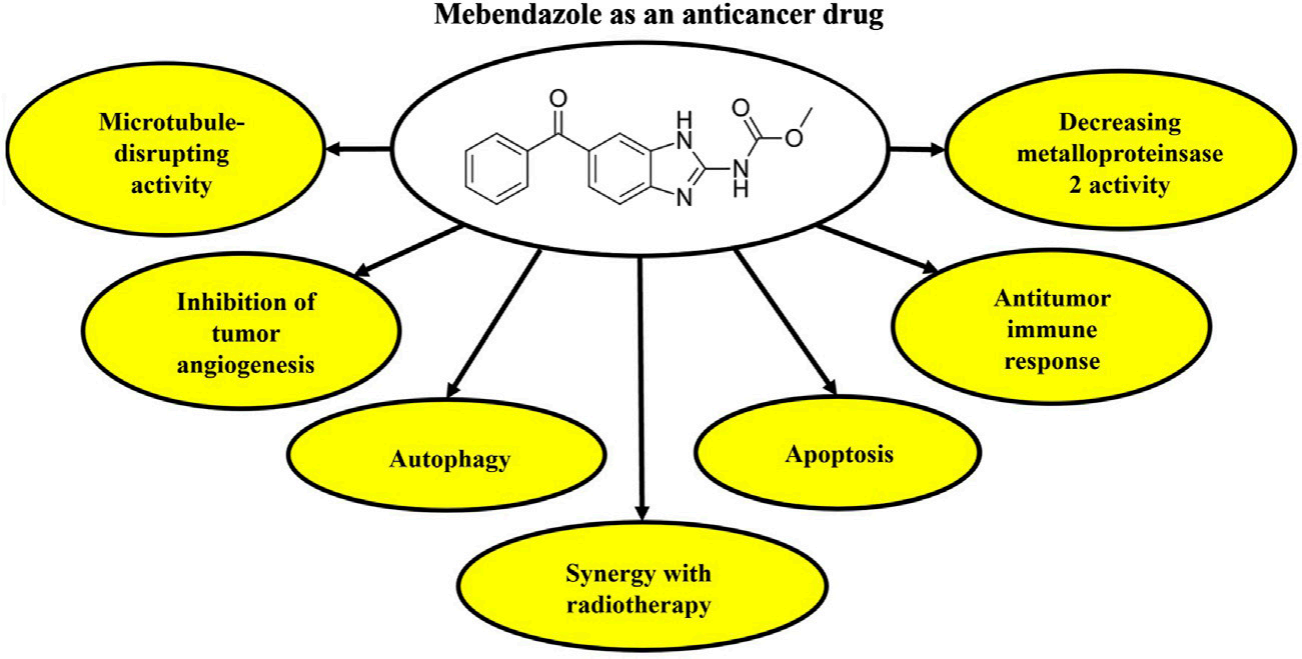
Anticancer activities of MBZ based on the mechanism of action. MBZ, mebendazole.

Several studies have provided evidence of the combined application of MBZ and radiation in reducing cell growth and angiogenesis, while increasing apoptosis in cancer cells (Guerini et al., 2019). A recent investigation has revealed that MBZ has the potential to modify DNA damage response proteins, thereby increasing the radiation sensitivity of cancer cells. In their study, co-treatment with MBZ and radiation increased γH2AXl as a marker of DNA damage in cancer cells. Overall, there is suggestive evidence to indicate that the simultaneous administration of MBZ and radiation induces a synergistic antagonism of the DNA damage response. Further investigations are warranted to substantiate the assertion that MBZ and radiation impede the development of microtubules, ultimately resulting in DNA impairment within neoplastic cells (Skibinski et al., 2018). Figure 1 presents the anticancer potential of MBZ.

## 3 MBZ against several cancer types: preclinical evidence

### 3.1 Breast cancer

The management of triple-negative breast cancer (TNBC) poses a challenge due to the lack of effective drugs. Accordingly, in an *in vitro* study, Zhang et al. showed that 0.7 µM of MBZ effectively increased the sensitivity of TNBC cells to radiation therapy. For this purpose, cells of TNBC were administered a singular dose of MBZ for 24 h before exposure to escalated levels of ionizing radiation. The results showed that MBZ at all tested radiation doses on MDA-MB-231 and SUM159PT cells leads to a significant increase in radiation sensitivity (Zhang et al., 2019).

In 2013, Coyne and colleagues evaluated the *in vitro* effectiveness of MBZ on the cell line resistant to breast cancer (SKBr-3). Based on the results, MBZ at a concentration of 0.5 μM decreased the survival of cancer cells by 63.1% (Coyne et al., 2013). Additionally, the combination of MBZ and methotrexate was evaluated to reduce the survival of breast cancer cells. Their results showed that the combination therapy of MBZ at doses of 1.5–100 µM and methotrexate (0.5–100 µM) exhibited synergistic effects against breast cancer cell lines (MDA-MB-231 and MCF-7) in an *in vitro* study. Upon administering MBZ, the mean reduction in cell viability for MCF-7 and MDA-MB-231 cells was found to be 81.3% and 66.8%, respectively (Alam et al., 2018).

### 3.2 Colon cancer

Chronic inflammation of the intestinal epithelium is a trigger for colorectal cancer (CRC). Typically, the dysregulation of prostaglandin-endoperoxide synthase 2 (PTGS2 or COX2) and the increased concentrations of prostaglandin E2 (PGE2) represent pathophysiological agents that facilitate the processes of inflammation and tumorigenesis (Guerini et al., 2019). In addition, VEGF/VEGFR2 pathways are activated during colon tumor progression. Based on previous reports, an *in vitro* study on platelets from people with colon cancer has shown that MBZ exhibits anti-CRC clinical activity through tubulin disruption and VEGFR2-mediated anti-angiogenic potential (Holler et al., 2012).

Williamson et al. confirmed that MBZ slowed down the growth of colon cancer xenografts and might be used as a way to prevent cancer in people who have a high chance of getting it. In a study conducted *in vivo*, it was observed that administering 35 mg/kg MBZ in combination with sulindac to ApcMin/+ mice resulted in a significant reduction in the occurrence of polyps by 90% compared to the group that received no treatment. Conversely, the use of sulindac alone did not prove to be an efficacious measure in preventing colon adenoma formation in ApcMin/+ mice. It may even lead to tumor formation in the colon (Williamson et al., 2016). In mice bearing colon cancer, MBZ significantly reduced the tumor volume (1,177 ± 1,109 mm^3^; *P* ≤ 0.001) and tumor weight (2.30 ± 1.97 g; *P* ≤ 0.0001) compared to the negative control group (weight 12.45 ± 2.0 g; volume 7,346 ± 1077 mm^3^). Additionally, MBZ increases mean survival time (MST) and the percentage of life span (ILS) in the animal study (51.2% ± 37% vs. 93%, respectively). This study suggests that MBZ strongly and selectively inhibits proliferation and induces apoptosis in colon cancer cells.

### 3.3 Gastric cancer

Gastric cancer ranks as the fourth most prevalent form of cancer and the second principal cause of cancer-related mortality worldwide (Jemal et al., 2011). Because gastric cancer is somewhat resistant to current clinical treatments, there is still no proven evidence-based treatment for such malignancy. The therapeutic approach for individuals diagnosed with gastric cancer involves the utilization of a treatment regimen consisting of 5-fluorouracil (5-FU)/cisplatin.; however, the toxicity of 5-FU, which leads to myelosuppression and gastrointestinal toxicity, may be a genuine and common issue for numerous cancer patients (Al-Batran et al., 2004).

Accordingly, in an *in vitro* study, Pinto et al. detailed the impacts of 5-FU combined with 0.15–20 μM of MBZ on a human ascites cell line derived from a gastric cancer tumor. MBZ is orally available and warrants further consideration as an anticancer agent due to its favorable pharmacokinetics, potential to enhance efficacy, and the significant safety profile of other drugs. Their finding demonstrated that MBZ/5-FU inhibited the proliferation of the malignant ascites cell line (i.e., AGP-01) derived from a primary intestinal-type gastric cancer. Additionally, their study showed the disruption of microtubule formation in AGP-01 cells treated with MBZ (Pinto et al., 2015).

### 3.4 Lung cancer

Non-small cell lung cancer (NSCLC) is the most frequent type of lung cancer that, despite early diagnosis, has a poor chance of treatment. Due to systemic metastases, chemotherapy is prescribed in more than 75% of patients with NSCLC. However, chemotherapy for such patients does not lead to improvement, even with high-rate treatment (Sasaki et al., 2002). Therefore, targeting molecular mediators sensitive to chemotherapy could be a promising strategy for finding a cure for certain types of cancer. Accordingly, tubulin, the major protein component of microtubules, is of great importance.

Sasaki et al. showed that MBZ exhibits a moderate level of spindle inhibition activity, yet it showcases a robust ability to combat tumors effectively. According to the results of their study, 0.1 µM of MBZ prevented the growth of human NSCLC *in vitro*. In this way, treatment of A549 and H460 human NSCLC cell lines with MBZ significantly suppressed cell proliferation. Moreover, MBZ induced depolymerization of tubulin and inhibited the formation of the normal spindle in NSCLC cells, resulting in mitotic arrest and subsequently apoptosis (Sasaki et al., 2002). Overall, another *in vitro* study demonstrated that a 0.165 µM dose of MBZ inhibited lung cancer cell growth 5-fold compared to control groups (Mukhopadhyay et al., 2002).

### 3.5 Medulloblastoma and glioma

*In vivo* studies showed that MBZ in doses of 25–50 mg/kg (oral) could suppress the Hh pathway in the DAOY human medulloblastoma cell line (Larsen et al., 2015). Additionally, Bai et al. demonstrated that MBZ could act as an angiogenesis inhibitor (50 mg/kg/day, administered orally in food) in the D425 human medulloblastoma cell line and murine parental or SMO-D477G mutated medulloblastoma (Bai et al., 2011). Another study showed that MBZ, as a single drug and even through synergistic effects with cisplatin, could result in the induction of apoptosis to increase CAL27 and inhibit SCC15 cells (IC_50_ values of 1.28 and 2.64 µM, respectively) (Zhang et al., 2017). De Witt et al. examined the viability suppression of GL261 murine glioma cells exposed to MBZ. Based on the results of their *in vitro* study, MBZ led to the depolymerization of microtubules (132 nM) followed by the induction of cell division arrest (192 nM) (De Witt et al., 2017). Additionally, Markowitz et al. showed that radiosensitization with MBZ (EC_50_ value of 35 nM) in murine GL261 glioma cells could lead to widespread anticancer effects *in vitro* (Markowitz et al., 2017).

### 3.6 Melanoma

XIAP is one of the most critical regulatory proteins for apoptosis, as it prevents cell death by inhibiting caspases 3, 7, and 9. Several investigations have demonstrated that enhanced expression of XIAP is associated with advanced disease progression stages in the context of melanoma (Daoud et al., 2022). Doudican et al. assessed the impact of MBZ on human melanoma cell lines, specifically M-14 and A-375. The research findings indicate that the utilization of MBZ resulted in a reduction of XIAP levels. This decrease in XIAP levels demonstrated a reciprocal relationship with the elevation of apoptotic markers, specifically PARP and caspase-9. They also established an M-14 xenograft model to evaluate the anticancer effect of 1 and 2 mg/kg doses of MBZ. MBZ inhibited tumor growth at the two doses tested without toxicity (83% and 77% inhibition, respectively) (Hiscutt et al., 2010; Doudican et al., 2013) (Table 1).

**TABLE 1:** *In vitro* and *in vivo* anticancer effects of MBZ against several cancer types.

Cancer type	*In vitro* studies	*In vivo* studies	Mechanism/outcome
Breast	0.5 µM–100 µM*;* SUM159PT and MDA-MB-231 TNBC cells Zhang et al. (2019)	10 or 20 mg/kg, i.p., 5 days/week for 3 weeks in mice Sensitizing triple-negative breast cancer (SUM159PT human TNBC) cells to ionizing radiation Zhang et al. (2019)	Increased the sensitivity to radiation therapy Markowitz et al. (2017) Synergistic effects with methotrexate Alam et al. (2018)
Colon	Less than 5 _µM; HT29, HCT-8 SW626, HCT 116, and RKO human colon cancer cells Nygren et al. (2013)	Reducing the number and size of tumor polyps alone and the synergistic effect with sulindac; SW480 or HT29 human colon cancer cells and mouse Williamson et al. (2016)	Cyclooxygenases 2 (COX2) inhibition Guerini et al. (2019) VEGFR2 phosphorylation Williamson et al. (2016) Inhibition of VEGF/VEGFR2 Guerini et al. (2019) Disruption of microtubule structure Peterson et al. (2012) Induction of anticancer activity in THP-1 monocyte Blom et al. (2019) BCR–ABL and BRAF kinases inhibition Nygren et al. (2013)
Gastric	0.15–20 μM; AGP-01ACP-02, ACP-03 human malignant ascites cell line derived from a primary gastric cancer tumor Pinto et al. (2015)	Not reported	MMP2 inhibitor Disruption of microtubule structure Inhibition of invasion and migration Pinto et al. (2015)
Lung	0.1 µM -A549, H1299, H460 Human Non-Small Cell Lung Cancer (Mukhopadhyay et al., 2002)	0.4, 0.8, and 1 mg/mouse/oral/day; dose-dependent inhibition of tumor growth; H460 and A549 human lung cancer Mukhopadhyay et al. (2002)	Induction of p53, p21 expression, and apoptosis Sasaki et al. (2002) Induced depolymerization of tubulin Inhibited the formation of normal spindle Larsen et al. (2015) VEGF and vascular cell adhesion protein 1 reduction Rubin et al. (2018)
Medulloblastoma and Glioma	132–192 nM A panel of 10 glioblastoma cell lines Bai et al. (2011)	25 and 50 mg/kg/day/oral in mice Reducing the growth of medulloblastoma cells Larsen et al. (2015) Increase in median survival time Larsen et al. (2015) 50 and 100 mg/kg/day/oral in mouse (glioma) Increase in median survival time De Witt et al. (2017)	Radiosensitization induction Markowitz et al. (2017) Angiogenesis inhibitor Bai et al. (2011) Hedgehog signaling inhibitor Larsen et al. (2015) Synergistic effects of cisplatin Zhang et al. (2017)
Melanoma	0.5 µM-Human melanoma M-14 and A-375 Doudican et al. (2008) 1 μmol/L M-14 and SK-Mel-19 chemoresistant melanoma cell lines Doudican et al. (2008)	Not reported	Induces apoptosis via Bcl-2 Inactivation and decrease in XIAP levels Doudican et al. (2008)
Ovarian cancer	0.25 µM, human ovarian cancer lines OVCAR8CR SKOV3CR SKOV3 and SKOV3CR	Not reported	Inhibited multiple cancer-related signal pathways, including ELK/SRF, MYC/MAX, E2F/DP1, and NF-κB; induced cell apoptosis, suppressed cell proliferation, and blunted tumor growth Huang et al. (2021)

## 4 Clinical trials of MBZ in cancer

To date, various clinical trials have been conducted to investigate the efficacy of MBZ as a potential treatment for cancer. In 2021, a single-center dose-escalation study of MBZ was conducted in 24 patients (18 with glioblastomas and 6 with anaplastic gliomas) in combination with temozolomide. The outcomes showed a mean survival duration of 21 months, with 41.7% of patients remaining alive at the end of 2 years, followed by 25% who survived at three and 4 years. For seventeen patients taking more than 1 month of MBZ from the date of diagnosis, median progression-free survival was 13.1 months (95% confidence interval [CI]: 8.8–14.6 months) but for 9.2 months (95% CI: 5.8–13.0 months) for seven patients received less than 1 month of MBZ.

Finally, MBZ at doses up to 200 mg/kg showed long-term safety and low toxicity (Gallia et al., 2021). During a Phase 2a clinical study, MBZ (up to 4 g/day and a serum concentration of 300 ng/mL) was administered to patients with advanced gastrointestinal cancer. In individuals with advanced cancer, the personalized dosage of MBZ was characterized by a favorable safety and tolerance profile (Mansoori et al., 2021). MBZ (200 mg/day) was employed in individuals diagnosed with high-grade glioma, who had recently begun undergoing temozolomide treatment (NCT01729260). Other clinical studies have also demonstrated the safety of MBZ at doses of up to 500 mg/day (NCT01173562). Other clinical trials are planned for recurrent or progressive pediatric brain tumors, a dose of 500 mg/day (NCT02644291), cirrhotic patients with advanced hepatocellular carcinoma at the dose of 100 mg/twice a day (NCT04443049), and the clinical safety and efficacy of MBZ in gastrointestinal cancer (NCT03628079).

## 5 MBZ drug delivery systems

Since pharmacokinetic limitations are key determinants of drug efficacy, new methods such as nanoformulation are being applied to overcome these obstacles (Fakhri et al., 2022a). MBZ has poor solubility and a disintegrating rate, thereby possessing lower bioavailability, which limits its effectiveness. To address these pharmacokinetic drawbacks, nanoformulations have been provided (Gigliobianco et al., 2018; Patel et al., 2024; Codina et al., 2025). In this way, MBZ conjugated with *N*-(2-hydroxypropyl)methacrylamide (HPMA)-based polymer carrier was shown to have anticancer impacts (Studenovský et al., 2021). In a study in 2022, MBZ-loaded folic acid-targeted chitosan nanoparticles (CS-FA-MBZ) decreased the number of hepatic cells that had been metastasized by murine mammary carcinoma cells in female BALB/c mice. Folic acid is used for the specific targeting of cancer cells due to their overexpression of the folate receptor on their membranes compared to normal cells. In addition, folic acid has the potential to be directly attached to polymeric nanoparticles. Additionally, CS-FA-MBZ showed the least adverse effect on cells and demonstrated higher efficacy in treating 4T1 TNBC. Also, CS-FA-MBZ exhibited suppressed impact on the growth of breast tumor cells, preventing the generation of metastasis and reducing the size of the tumor cells. This formulation also reduced the adverse effects of MBZ, which causes a little elevation in liver enzymes in TNBC BALB/c mice (Kefayat et al., 2022).

In another study, MBZ in combination with 30% of squalene, compritol 888 ATO, and pluronic F68 in sizes between 300 and 600 nm in the form of nanostructured lipid formulations (NLF) particles, showed the best potential for loading MBZ in NLF and have anticancer effects (Graves et al., 2015). In another study, the electrochemical response of MBZ was evaluated using carbon nanostructured modified glassy carbon electrodes, which had been modified with carbon nanotubes. It showed a better voltammetric response of MBZ for the treatment of various cancers (Ghalkhani et al., 2016).

MBZ nanosuspension demonstrated high stability and effectiveness in drug distribution via using the media milling method in a well-plate setup and a glass-vial-based device in a planetary mill (Van Eerdenbrugh et al., 2009). MBZ re-dispersible microparticles (RDM) carried by low-substituted hydroxypropylcellulose (L-HPC) showed improved solubility and a better distribution of the drug in a murine model of infection with the nematode parasite *Trichinella spiralis*.

MBZ-loaded mixed micelles (MBZ-loaded MMs) suppressed the vascular endothelial growth factor, which plays a vital role in the progression of endothelial cells in various cancers. They improved the distribution of MBZ (Elmaaty et al., 2022). Overall, novel delivery systems of MBZ could potentiate its therapeutic effects against cancer.

## 6 Conclusion

**FIGURE 2:**
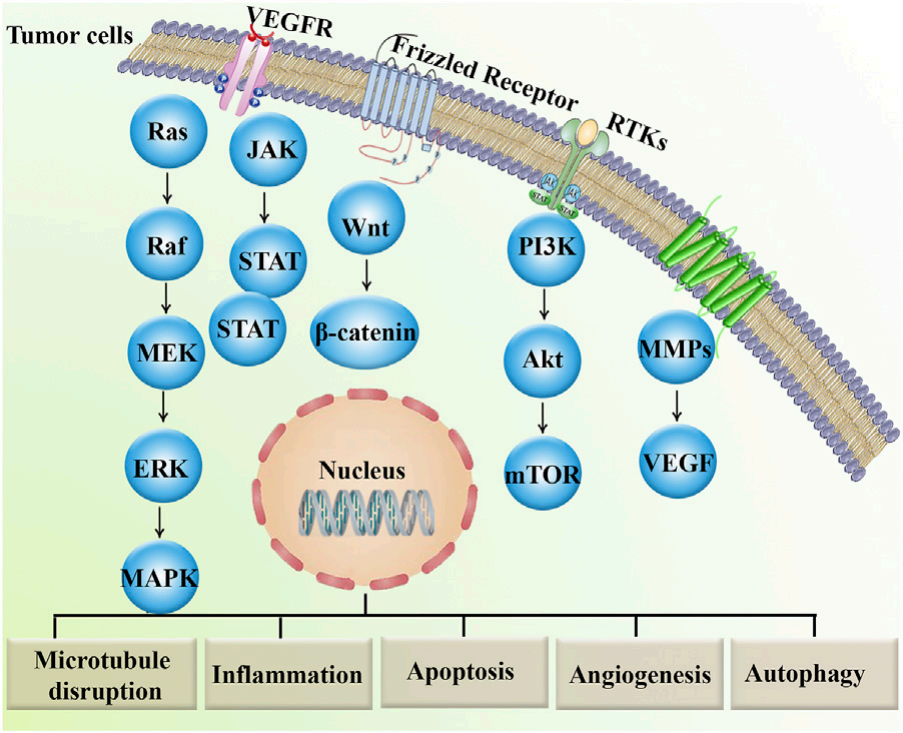
Major dysregulated signaling pathways involved in cancer drug resistance.Akt, protein kinase B; ERK, extracellular signal-regulated kinase; JAK, janus kinase; MAPKs, mitogen-activated protein kinases; MEK, MAP–ERK kinase; mTOR, mammalian target of rapamycin; MMPs, matrix metalloproteinases; PI3K, phosphoinositide 3-kinase; RTK, receptor tyrosine kinase; STAT, signal transducer and activator of transcription; TNF, tumor necrosis factor; VEGF, vascular endothelial growth factor; VEGFR, vascular endothelial growth factor receptor.

In conclusion, *in vitro*, *in vivo,* and clinical trial data provided ample evidence of the anticancer effect of MBZ treatment (Figure 1) and its related role in preventing cancer drug resistance mediators (Figure 2).

**FIGURE 3:**
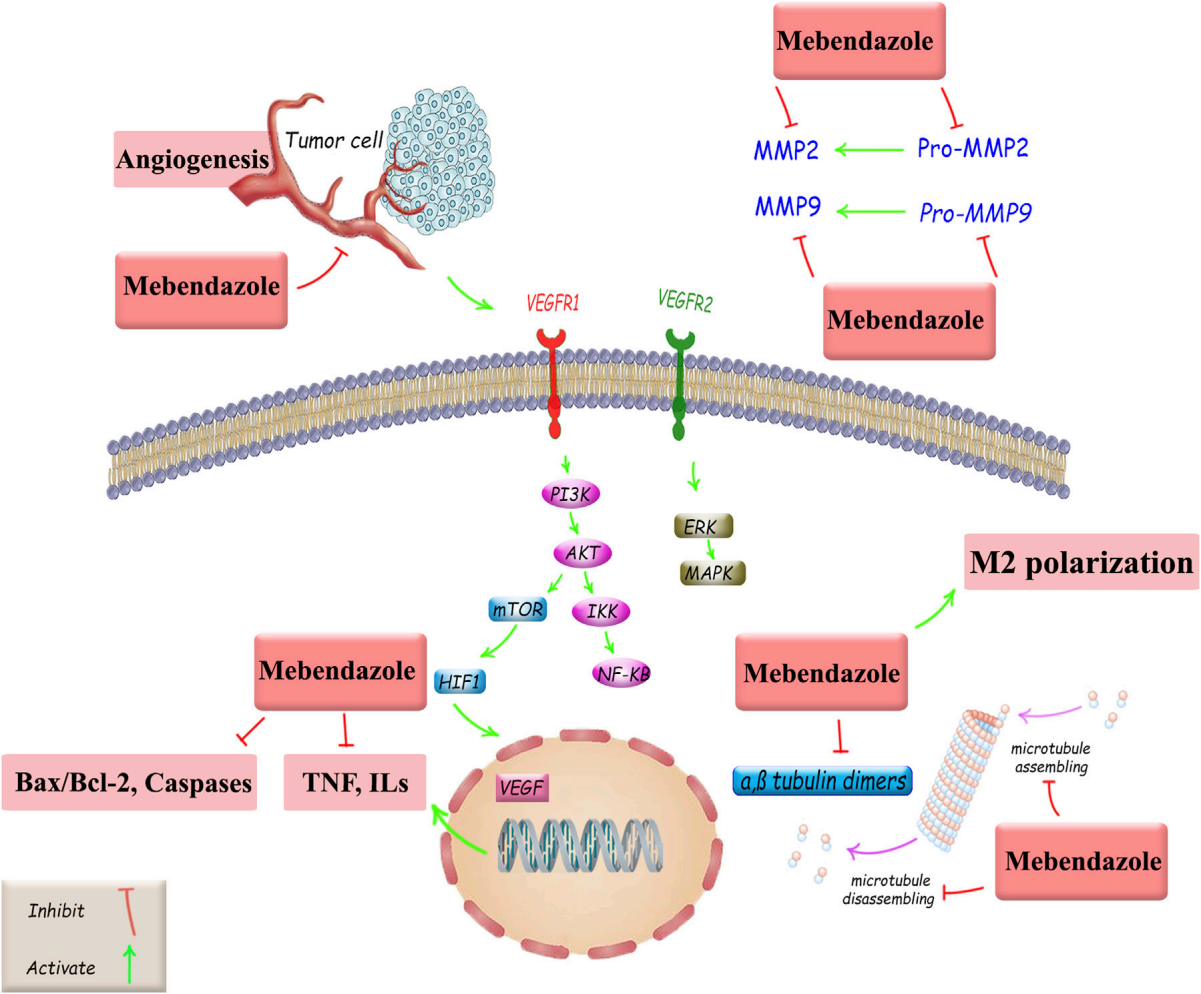
Anticancer mechanisms of MBZ and related therapeutic targets against cancer drug resistance. Akt, protein kinase B; Bax, Bcl-2-associated X protein; Bcl-2, B-cell leukemia/lymphoma; ERK, extracellular signal-regulated kinase; HIF-1, hypoxia-inducible factor 1; ILs, interleukins, MAPKs, mitogen-activated protein kinases; MMP, matrix metalloproteinase; mTOR, mammalian target of rapamycin; NF-κB, nuclear factor kappa B; PI3K, phosphoinositide 3-kinase; TNF, tumor necrosis factor; VEGF, vascular endothelial growth factor; VEGFR, vascular endothelial growth factor receptor.

This low-cost drug, characterized by completely stable pharmacokinetics and low toxicity (Son et al., 2020; Choi et al., 2021; Meco et al., 2023), is a suitable and powerful option for drug repositioning in cancer treatment, both alone and in combination with existing standard therapies. However, other critical approaches are also employed in oncology drug repositioning, including molecular docking, genetic association, pathway mapping, signature matching, retrospective clinical analysis, novel data sources, phenotypic screening, and binding assays (Pushpakom et al., 2019). In this study, we investigated MBZ in terms of its cancer treatment mechanisms and its role in treating various cancers through the regulation of tumor angiogenesis, autophagy, and apoptosis, as well as the modulation of effective signaling pathways, induction of antitumor immune responses, and suppression of MMP2 activity. Such mechanisms are followed by the main anticancer potential of MBZ on microtubule targeting. Despite the similar mechanisms of MBZ and colchicine in targeting microtubules, they exhibit distinct selectivity, cellular uptake, and pharmacokinetics. Accordingly, possessing a higher volume of distribution makes a multi-organ toxicity profile for colchicine (Lu et al., 2012; Cai et al., 2025). Although we have now demonstrated diverse and interesting findings on the effects of MB on various cancers, further research is needed in the future for its clinical application (Figure 3).

The findings of the present review indicate that the practical application of MBZ in anticancer treatments will expand. Further understanding of the anticancer mechanisms of MBZ will pave the way for identifying the novel clinical relevance of MBZ as an anticancer agent. Prospective research avenues should encompass comprehensive *in vitro* and *in vivo* investigations aimed at elucidating the exact signaling pathways of MBZ, followed by tightly regulated clinical trials. The proposed investigation seeks to explore the potential anticancer properties of MBZ in the areas of prophylaxis, mitigation, and therapeutic intervention for a diverse range of malignancies. Despite the promising findings on MBZ oncology repositioning, some challenges and limitations remained, including poor solubility and a slow disintegrating rate, which resulted in lower bioavailability. Furthermore, the variety of resistance mechanisms and variability in response to different tumor types, due to differences in genetic profiles, tumor microenvironments, and drug metabolism between patients, raises concerns about MBZ’s anticancer potential. These pharmacokinetic limitations urge the need to develop novel formulations for MBZ, such as polymeric nanoparticles, nanosuspensions, solid lipid nanoparticles, and micelles. To address the limitations mentioned above, combination therapies and patient-specific approaches will be critical for extrapolating MBZ’s anticancer effects into clinical success.

## Glossary

Akt: protein kinase B
AP1: activator protein-1
ATAT: α-tubulin *N*-acetyl transferase
ATG5: autophagy gene 5
ATG7: autophagy gene 7
ATM: ataxia telangiectasia-mutated
Bax: Bcl-2-associated X protein
Bcl-2: B-cell leukemia/lymphoma
Bcl-XL: B-cell lymphoma-extra large
CRC: colorectal cancer
DYRK1b: dual specificity tyrosine-phosphorylation-regulated kinase 1B
ECs: endothelial cells
ELK1: E-26-like protein 1
ERK1/2: extracellular signal-regulated kinase 1/2
GLI: glioma-associated oncogene
Hh: Hedgehog
HIF-1: hypoxia-inducible factor 1
IL-6: interleukin 6
IL-8: interleukin 8
JAK: Janus kinase
L-HPC: low-substituted hydroxypropylcellulose
MAPKs: mitogen-activated protein kinases
MAX: myc-associated factor X
MBZ: Mebendazole
MEK: MAP–ERK kinase
MM: mixed micelles
MMP: matrix metalloproteinase
mTOR: mammalian target of rapamycin
NF-κB: nuclear factor kappa B
MYC: c-myc proto-oncogene
NLF: nanostructured lipid formulations
NSCLC: Non-small cell lung cancer
p53: tumor protein p53
PARP: ADP-ribose polymerase
PBMCs: peripheral blood mononuclear cells
PGE2: prostaglandin E2
PI3K: phosphoinositide 3-kinase
PLCγ: phospholipase C gamma
PTCH1: patched 1
PTGS2: prostaglandin-Endoperoxide Synthase 2
RDM: re-dispersible microparticles
SMO: smoothened
STAT1/2: signal transducer and activator of transcription 1 and 2
SRF: serum response factor
TFEB: transcription factor EB
TNBC: triple-negative breast cancer
TNF: tumor necrosis factor
VEGF: vascular endothelial growth factor
VEGFR2: vascular endothelial growth factor receptor-2
XIAP: X-linked apoptosis inhibitor.

## References

[B1] Alam S. M. Asad F. Shams H. (2018). Mebendazole inexplicably reducing the breast cancer cells viability preclinically by incitement effects with methotrexate. RADS J. Pharm. Pharm. Sci. 6, 101–106.

[B2] Al-Batran S.-E. Atmaca A. Hegewisch-Becker S. Jaeger D. Hahnfeld S. Rummel M. J. (2004). Phase II trial of biweekly infusional fluorouracil, folinic acid, and oxaliplatin in patients with advanced gastric cancer. J. Clin. Oncol. 22, 658–663. 10.1200/JCO.2004.07.042 14966088

[B3] Andersson C. R. Selvin T. Blom K. Rubin J. Berglund M. Jarvius M. (2020). Mebendazole is unique among tubulin-active drugs in activating the MEK–ERK pathway. Sci. Rep. 10, 13124. 10.1038/s41598-020-68986-0 32753665 PMC7403428

[B4] Aras S. Zaidi M. R. (2017). TAMeless traitors: macrophages in cancer progression and metastasis. Br. J. Cancer 117, 1583–1591. 10.1038/bjc.2017.356 29065107 PMC5729447

[B5] Baghban N. Ullah M. Nabipour I. (2023). The current trend of exosome in epithelial ovarian cancer studies: a bibliometric review. Front. Pharmacol. 14, 1082066. 10.3389/fphar.2023.1082066 36969852 PMC10034012

[B6] Bai R.-Y. Staedtke V. Aprhys C. M. Gallia G. L. Riggins G. J. (2011). Antiparasitic mebendazole shows survival benefit in 2 preclinical models of glioblastoma multiforme. Neuro. Oncol. 13, 974–982. 10.1093/neuonc/nor077 21764822 PMC3158014

[B7] Bai R.-Y. Staedtke V. Rudin C. M. Bunz F. Riggins G. J. (2015). Effective treatment of diverse medulloblastoma models with mebendazole and its impact on tumor angiogenesis. Neuro. Oncol. 17, 545–554. 10.1093/neuonc/nou234 25253417 PMC4483072

[B8] Belloni D. Veschini L. Foglieni C. Dell’Antonio G. Caligaris-Cappio F. Ferrarini M. (2010). Bortezomib induces autophagic death in proliferating human endothelial cells. Exp. Cell Res. 316, 1010–1018. 10.1016/j.yexcr.2009.11.005 19917281

[B9] Blom K. Rubin J. Berglund M. Jarvius M. Lenhammar L. Parrow V. (2019). Mebendazole-induced M1 polarisation of THP-1 macrophages may involve DYRK1B inhibition. BMC Res. Notes 12, 234. 10.1186/s13104-019-4273-5 31010428 PMC6477744

[B10] Bold R. J. Termuhlen P. M. McConkey D. J. (1997). Apoptosis, cancer and cancer therapy. Surg. Oncol. 6, 133–142. 10.1016/S0960-7404(97)00015-7 9576629

[B11] Cai J. He M. Wang Y. Zhang H. Xu Y. Wang Y. (2025). Discovery of a novel microtubule destabilizing agent targeting the colchicine site based on molecular docking. Biochem. Pharmacol. 234, 116804. 10.1016/j.bcp.2025.116804 39956210

[B12] Chai J.-Y. Jung B.-K. Hong S.-J. (2021). Albendazole and mebendazole as anti-parasitic and anti-cancer agents: an update. Korean J. Parasitol. 59, 189–225. 10.3347/kjp.2021.59.3.189 34218593 PMC8255490

[B13] Chauhan S. Ahmed Z. Bradfute S. B. Arko-Mensah J. Mandell M. A. Won Choi S. (2015). Pharmaceutical screen identifies novel target processes for activation of autophagy with a broad translational potential. Nat. Commun. 6, 8620. 10.1038/ncomms9620 26503418 PMC4624223

[B14] Choi H. S. Ko Y. S. Jin H. Kang K. M. Ha I. B. Jeong H. (2021). Anticancer effect of benzimidazole derivatives, especially mebendazole, on triple-negative breast cancer (TNBC) and radiotherapy-resistant TNBC *in vivo* and *in vitro* . Molecules 26, 5118. 10.3390/molecules26175118 34500557 PMC8433818

[B15] Choi H. S. Ko Y. S. Jin H. Kang K. M. Ha I. B. Jeong H. (2022). Mebendazole increases anticancer activity of radiotherapy in radiotherapy-resistant triple-negative breast cancer cells by enhancing natural killer cell-mediated cytotoxicity. Int. J. Mol. Sci. 23, 15493. 10.3390/ijms232415493 36555137 PMC9779603

[B16] Codina A. V. Indelman P. Hinrichsen L. I. Lamas M. C. (2025). Significant improvement in bioavailability and therapeutic efficacy of mebendazole oral nano-systems assessed in a murine model with extreme phenotypes of susceptibility to *Trichinella spiralis* . Preprints. 10.20944/preprints202504.2256.v1 PMC12389038 40871089

[B17] Coyne C. P. Jones T. Bear R. (2013). Gemcitabine-(C4-amide)-[anti-HER2/neu] anti-neoplastic cytotoxicity in dual combination with mebendazole against chemotherapeutic-resistant mammary adenocarcinoma. J. Clin. Exp. Oncol. 2, 1000109. Available online at: http://www.ncbi.nlm.nih.gov/pubmed/26251840. 26251840 PMC4531380

[B18] Daoud M. Broxtermann P. N. Schorn F. Werthenbach J. P. Seeger J. M. Schiffmann L. M. (2022). XIAP promotes melanoma growth by inducing tumour neutrophil infiltration. EMBO Rep. 23, e53608. 10.15252/embr.202153608 35437868 PMC9171690

[B19] Das C. K. Mandal M. Kögel D. (2018). Pro-survival autophagy and cancer cell resistance to therapy. Cancer Metastasis Rev. 37, 749–766. 10.1007/s10555-018-9727-z 29536228

[B20] De Witt M. Gamble A. Hanson D. Markowitz D. Powell C. Al Dimassi S. (2017). Repurposing mebendazole as a replacement for vincristine for the treatment of brain tumors. Mol. Med. 23, 50–56. 10.2119/molmed.2017.00011 28386621 PMC5403762

[B21] Dong D. Yu X. Xu J. Yu N. Liu Z. Sun Y. (2024). Cellular and molecular mechanisms of gastrointestinal cancer liver metastases and drug resistance. Drug resist. updat. 77, 101125. 10.1016/j.drup.2024.101125 39173439

[B22] Dorléans A. Gigant B. Ravelli R. B. G. Mailliet P. Mikol V. Knossow M. (2009). Variations in the colchicine-binding domain provide insight into the structural switch of tubulin. Proc. Natl. Acad. Sci. 106, 13775–13779. 10.1073/pnas.0904223106 19666559 PMC2728970

[B23] Doudican N. Rodriguez A. Osman I. Orlow S. J. (2008). Mebendazole induces apoptosis via bcl-2 inactivation in chemoresistant melanoma cells. Mol. Cancer Res. 6, 1308–1315. 10.1158/1541-7786.MCR-07-2159 18667591

[B24] Doudican N. A. Byron S. A. Pollock P. M. Orlow S. J. (2013). XIAP downregulation accompanies mebendazole growth inhibition in melanoma xenografts. Anticancer. Drugs 24, 181–188. 10.1097/CAD.0b013e32835a43f1 23059386

[B25] Elmaaty A. A. Darwish K. M. Chrouda A. Boseila A. A. Tantawy M. A. Elhady S. S. (2022). *In silico* and *in vitro* studies for benzimidazole anthelmintics repurposing as VEGFR-2 antagonists: novel mebendazole-loaded mixed micelles with enhanced dissolution and anticancer activity. ACS Omega 7, 875–899. 10.1021/acsomega.1c05519 35036753 PMC8757357

[B26] Fakhri S. Abdian S. Zarneshan S. N. Moradi S. Z. Farzaei M. H. Abdollahi M. (2022a). Nanoparticles in combating neuronal dysregulated signaling pathways: recent approaches to the nanoformulations of phytochemicals and synthetic drugs against neurodegenerative diseases. Int. J. Nanomedicine 17, 299–331. 10.2147/IJN.S347187 35095273 PMC8791303

[B27] Fakhri S. Moradi S. Z. Faraji F. Farhadi T. Hesami O. Iranpanah A. (2023). Current advances in nanoformulations of therapeutic agents targeting tumor microenvironment to overcome drug resistance. Cancer Metastasis Rev. 42, 959–1020. 10.1007/s10555-023-10119-w 37505336

[B28] Fakhri S. Moradi S. Z. Farzaei M. H. Bishayee A. (2022b). Modulation of dysregulated cancer metabolism by plant secondary metabolites: a mechanistic review. Semin. Cancer Biol. 80, 276–305. 10.1016/j.semcancer.2020.02.007 32081639

[B29] Fan M. Chambers T. C. (2001). Role of mitogen-activated protein kinases in the response of tumor cells to chemotherapy. Drug resist. updat. 4, 253–267. 10.1054/drup.2001.0214 11991680

[B30] Foster K. E. Burland T. G. Gull K. (1987). A mutant beta-tubulin confers resistance to the action of benzimidazole-carbamate microtubule inhibitors both *in vivo* and *in vitro* . Eur. J. Biochem. 163, 449–455. 10.1111/j.1432-1033.1987.tb10890.x 3830165

[B31] Gallia G. L. Holdhoff M. Brem H. Joshi A. D. Hann C. L. Bai R.-Y. (2021). Mebendazole and temozolomide in patients with newly diagnosed high-grade gliomas: results of a phase 1 clinical trial. Neuro-Oncology Adv. 3, vdaa154. 10.1093/noajnl/vdaa154 PMC7817892 33506200

[B32] Gao J. Zhao Y. Lv Y. Chen Y. Wei B. Tian J. (2013). Mirk/Dyrk1B mediates G0/G1 to S phase cell cycle progression and cell survival involving MAPK/ERK signaling in human cancer cells. Cancer Cell Int. 13, 2. 10.1186/1475-2867-13-2 23311607 PMC3575355

[B33] Ghalkhani M. Beheshtian J. Salehi M. (2016). Electrochemical and DFT study of an anticancer and active anthelmintic drug at carbon nanostructured modified electrode. Mater. Sci. Eng. C 69, 1345–1353. 10.1016/j.msec.2016.08.031 27612835

[B34] Gigliobianco M. R. Casadidio C. Censi R. Di Martino P. (2018). Nanocrystals of poorly soluble drugs: drug bioavailability and physicochemical stability. Pharmaceutics 10, 134. 10.3390/pharmaceutics10030134 30134537 PMC6161002

[B35] Graves R. A. Ledet G. A. Nation C. A. Pramar Y. V. Bostanian L. A. Mandal T. K. (2015). Effect of squalane on mebendazole-loaded Compritol ® nanoparticles. J. Biomater. Sci. Polym. Ed. 26, 868–880. 10.1080/09205063.2015.1061351 26062393 PMC4685693

[B36] Guerini T. Maddalo B. Frassine B. Bonù M. L. Frassine F. Baiguini A. (2019). Mebendazole as a candidate for drug repurposing in oncology: an extensive review of current literature. Cancers (Basel) 11, 1284. 10.3390/cancers11091284 31480477 PMC6769799

[B37] Hein A. L. Oullette M. M. Yan Y. (2014). Radiation-induced signaling pathways that promote cancer cell survival. Int. J. Oncol. 45, 1813–1819. 10.3892/ijo.2014.2614 25174607 PMC4203326

[B38] Hiscutt E. L. Hill D. S. Martin S. Kerr R. Harbottle A. Birch-Machin M. (2010). Targeting X-linked inhibitor of apoptosis protein to increase the efficacy of endoplasmic reticulum stress-induced apoptosis for melanoma therapy. J. Invest. Dermatol. 130, 2250–2258. 10.1038/jid.2010.146 20520630

[B39] Holler J. G. Christensen S. B. Slotved H.-C. Rasmussen H. B. Guzman A. Olsen C.-E. (2012). Novel inhibitory activity of the *Staphylococcus aureus* NorA efflux pump by a kaempferol rhamnoside isolated from Persea lingue Nees. J. Antimicrob. Chemother. 67, 1138–1144. 10.1093/jac/dks005 22311936

[B40] Huang L. Zhao L. Zhang J. He F. Wang H. Liu Q. (2021). Antiparasitic mebendazole (MBZ) effectively overcomes cisplatin resistance in human ovarian cancer cells by inhibiting multiple cancer-associated signaling pathways. Aging (Albany. NY) 13, 17407–17427. 10.18632/aging.203232 34232919 PMC8312413

[B41] Jacks T. Weinberg R. A. (2002). Taking the study of cancer cell survival to a new dimension. Cell 111, 923–925. 10.1016/S0092-8674(02)01229-1 12507419

[B42] Jain S. K. Parsanathan R. Levine S. N. Bocchini J. A. Holick M. F. Vanchiere J. A. (2020). The potential link between inherited G6PD deficiency, oxidative stress, and vitamin D deficiency and the racial inequities in mortality associated with COVID-19. Free Radic. Biol. Med. 161, 84–91. 10.1016/j.freeradbiomed.2020.10.002 33038530 PMC7539020

[B43] Jemal A. Bray F. Center M. M. Ferlay J. Ward E. Forman D. (2011). Global cancer statistics. Ca. Cancer J. Clin. 61, 69–90. 10.3322/caac.20107 21296855

[B44] Joe N. S. Godet I. Milki N. Ain N. U. I. Oza H. H. Riggins G. J. (2022). Mebendazole prevents distant organ metastases in part by decreasing ITGβ4 expression and cancer stemness. Breast Cancer Res. 24, 98. 10.1186/s13058-022-01591-3 36578038 PMC9798635

[B45] Jordan M. A. Wilson L. (1998). Microtubules and actin filaments: dynamic targets for cancer chemotherapy. Curr. Opin. Cell Biol. 10, 123–130. 10.1016/S0955-0674(98)80095-1 9484604

[B46] Kamal A. Shaik A. B. Polepalli S. Kumar G. B. Reddy V. S. Mahesh R. (2015). Synthesis of arylpyrazole linked benzimidazole conjugates as potential microtubule disruptors. Bioorg. Med. Chem. 23, 1082–1095. 10.1016/j.bmc.2015.01.004 25648686

[B47] Kardideh B. Samimi Z. Norooznezhad F. Kiani S. Mansouri K. (2019). Autophagy, cancer and angiogenesis: where is the link? Cell Biosci. 9, 65. 10.1186/s13578-019-0327-6 31428311 PMC6693242

[B48] Kefayat A. Hosseini M. Ghahremani F. Jolfaie N. A. Rafienia M. (2022). Biodegradable and biocompatible subcutaneous implants consisted of pH-sensitive mebendazole-loaded/folic acid-targeted chitosan nanoparticles for murine triple-negative breast cancer treatment. J. Nanobiotechnology 20, 169. 10.1186/s12951-022-01380-2 35361226 PMC8973744

[B49] Kim E. K. Choi E.-J. (2010). Pathological roles of MAPK signaling pathways in human diseases. Biochim. Biophys. Acta - Mol. Basis Dis. 1802, 396–405. 10.1016/j.bbadis.2009.12.009 20079433

[B50] Kumar P. Jagtap Y. A. Patwa S. M. Kinger S. Dubey A. R. Prajapati V. K. (2022). Autophagy based cellular physiological strategies target oncogenic progression. J. Cell. Physiol. 237, 258–277. 10.1002/jcp.30567 34448206

[B51] Kumar S. Guru S. K. Pathania A. S. Kumar A. Bhushan S. Malik F. (2013). Autophagy triggered by magnolol derivative negatively regulates angiogenesis. Cell Death Dis. 4, e889. 10.1038/cddis.2013.399 24176847 PMC3920944

[B52] Larsen A. R. Bai R.-Y. Chung J. H. Borodovsky A. Rudin C. M. Riggins G. J. (2015). Repurposing the antihelmintic mebendazole as a hedgehog inhibitor. Mol. Cancer Ther. 14, 3–13. 10.1158/1535-7163.MCT-14-0755-T 25376612 PMC4297232

[B53] Lu Y. Chen J. Xiao M. Li W. Miller D. D. (2012). An overview of tubulin inhibitors that interact with the colchicine binding site. Pharm. Res. 29, 2943–2971. 10.1007/s11095-012-0828-z 22814904 PMC3667160

[B54] Mansoori S. Fryknäs M. Alvfors C. Loskog A. Larsson R. Nygren P. (2021). A phase 2a clinical study on the safety and efficacy of individualized dosed mebendazole in patients with advanced gastrointestinal cancer. Sci. Rep. 11, 8981. 10.1038/s41598-021-88433-y 33903692 PMC8076239

[B55] Markowitz D. Ha G. Ruggieri R. Symons M. (2017). Microtubule-targeting agents can sensitize cancer cells to ionizing radiation by an interphase-based mechanism. Onco. Targets. Ther. 10, 5633–5642. 10.2147/OTT.S143096 29200877 PMC5703169

[B56] Meco D. Attinà G. Mastrangelo S. Navarra P. Ruggiero A. (2023). Emerging perspectives on the antiparasitic mebendazole as a repurposed drug for the treatment of brain cancers. Int. J. Mol. Sci. 24, 1334. 10.3390/ijms24021334 36674870 PMC9862092

[B57] Mondal S. Adhikari N. Banerjee S. Amin S. A. Jha T. (2020). Matrix metalloproteinase-9 (MMP-9) and its inhibitors in cancer: a minireview. Eur. J. Med. Chem. 194, 112260. 10.1016/j.ejmech.2020.112260 32224379

[B58] Mudduluru G. Walther W. Kobelt D. Dahlmann M. Treese C. Assaraf Y. G. (2016). Repositioning of drugs for intervention in tumor progression and metastasis: old drugs for new targets. Drug resist. updat. 26, 10–27. 10.1016/j.drup.2016.03.002 27180307

[B59] Mukhopadhyay T. Sasaki J. Ramesh R. Roth J. A. (2002). Mebendazole elicits a potent antitumor effect on human cancer cell lines both *in vitro* and *in vivo* . Clin. Cancer Res. 8, 2963–2969. Available online at: http://www.ncbi.nlm.nih.gov/pubmed/12231542. 12231542

[B60] Murray P. J. (2017). Macrophage polarization. Annu. Rev. Physiol. 79, 541–566. 10.1146/annurev-physiol-022516-034339 27813830

[B61] Mutschelknaus L. Peters C. Winkler K. Yentrapalli R. Heider T. Atkinson M. J. (2016). Exosomes derived from squamous head and neck cancer promote cell survival after ionizing radiation. PLoS One 11, e0152213. 10.1371/journal.pone.0152213 27006994 PMC4805173

[B62] Nguyen T. M. B. Subramanian I. V. Xiao X. Ghosh G. Nguyen P. Kelekar A. (2009). Endostatin induces autophagy in endothelial cells by modulating Beclin 1 and β-catenin levels. J. Cell. Mol. Med. 13, 3687–3698. 10.1111/j.1582-4934.2009.00722.x 19298526 PMC4516517

[B63] Nunes M. Bartosch C. Abreu M. H. Richardson A. Almeida R. Ricardo S. (2024). Deciphering the molecular mechanisms behind drug resistance in ovarian cancer to unlock efficient treatment options. Cells 13, 786. 10.3390/cells13090786 38727322 PMC11083313

[B64] Nygren P. Fryknäs M. Ågerup B. Larsson R. (2013). Repositioning of the anthelmintic drug mebendazole for the treatment for colon cancer. J. Cancer Res. Clin. Oncol. 139, 2133–2140. 10.1007/s00432-013-1539-5 24135855 PMC3825534

[B65] Oprea T. I. Mestres J. (2012). Drug repurposing: far beyond new targets for old drugs. AAPS J. 14, 759–763. 10.1208/s12248-012-9390-1 22826034 PMC3475856

[B66] Pantziarka P. Bouche G. Meheus L. Sukhatme V. Sukhatme V. P. (2014). Repurposing drugs in oncology (ReDO)-mebendazole as an anti-cancer agent. Ecancermedicalscience 8, 443. 10.3332/ecancer.2014.443 25075217 PMC4096024

[B67] Patel R. D. Patel A. S. Patel H. J. Sarvepalli S. Patel K. (2024). Development of rapidly soluble mebendazole nanosuspension for colorectal cancer. J. Drug Deliv. Sci. Technol. 91, 105276. 10.1016/j.jddst.2023.105276

[B68] Petersen J. S. S. M. Baird S. K. (2021). Treatment of breast and colon cancer cell lines with anti-helmintic benzimidazoles mebendazole or albendazole results in selective apoptotic cell death. J. Cancer Res. Clin. Oncol. 147, 2945–2953. 10.1007/s00432-021-03698-0 34148157 PMC11802096

[B69] Peterson J. E. Zurakowski D. Italiano J. E. Michel L. V. Connors S. Oenick M. (2012). VEGF, PF4 and PDGF are elevated in platelets of colorectal cancer patients. Angiogenesis 15, 265–273. 10.1007/s10456-012-9259-z 22402885

[B70] Pietras K. Pahler J. Bergers G. Hanahan D. (2008). Functions of paracrine PDGF signaling in the proangiogenic tumor stroma revealed by pharmacological targeting. PLoS Med. 5, e19. 10.1371/journal.pmed.0050019 18232728 PMC2214790

[B71] Pinto L. C. Soares B. M. Pinheiro J. de J. V. Riggins G. J. Assumpção P. P. Burbano R. M. R. (2015). The anthelmintic drug mebendazole inhibits growth, migration and invasion in gastric cancer cell model. Toxicol. Vitr. 29, 2038–2044. 10.1016/j.tiv.2015.08.007 26315676

[B72] Pushpakom S. Iorio F. Eyers P. A. Escott K. J. Hopper S. Wells A. (2019). Drug repurposing: progress, challenges and recommendations. Nat. Rev. Drug Discov. 18, 41–58. 10.1038/nrd.2018.168 30310233

[B73] Ravelli R. B. G. Gigant B. Curmi P. A. Jourdain I. Lachkar S. Sobel A. (2004). Insight into tubulin regulation from a complex with colchicine and a stathmin-like domain. Nature 428, 198–202. 10.1038/nature02393 15014504

[B74] Rubin J. Mansoori S. Blom K. Berglund M. Lenhammar L. Andersson C. (2018). Mebendazole stimulates CD14+ myeloid cells to enhance T-cell activation and tumour cell killing. Oncotarget 9, 30805–30813. 10.18632/oncotarget.25713 30112108 PMC6089388

[B75] Rushworth L. K. Hewit K. Munnings-Tomes S. Somani S. James D. Shanks E. (2020). Repurposing screen identifies mebendazole as a clinical candidate to synergise with docetaxel for prostate cancer treatment. Br. J. Cancer 122, 517–527. 10.1038/s41416-019-0681-5 31844184 PMC7028732

[B76] Sasaki J. Ramesh R. Chada S. Gomyo Y. Roth J. A. Mukhopadhyay T. (2002). The anthelmintic drug mebendazole induces mitotic arrest and apoptosis by depolymerizing tubulin in non-small cell lung cancer cells. Mol. Cancer Ther. 1, 1201–1209. Available online at: http://www.ncbi.nlm.nih.gov/pubmed/12479701. 12479701

[B77] Satija G. Sharma B. Madan A. Iqubal A. Shaquiquzzaman M. Akhter M. (2022). Benzimidazole based derivatives as anticancer agents: structure activity relationship analysis for various targets. J. Heterocycl. Chem. 59, 22–66. 10.1002/jhet.4355

[B78] Sawyers C. (2004). Targeted cancer therapy. Nature 432, 294–297. 10.1038/nature03095 15549090

[B79] Shrivastava N. Naim M. J. Alam M. J. Nawaz F. Ahmed S. Alam O. (2017). Benzimidazole scaffold as anticancer agent: synthetic approaches and structure–activity relationship. Arch. Pharm. Weinh. 350, 350. 10.1002/ardp.201700040 28544162

[B80] Skibinski C. G. Williamson T. Riggins G. J. (2018). Mebendazole and radiation in combination increase survival through anticancer mechanisms in an intracranial rodent model of malignant meningioma. J. Neurooncol. 140, 529–538. 10.1007/s11060-018-03009-7 30414098 PMC6387633

[B81] Sleire L. Førde H. E. Netland I. A. Leiss L. Skeie B. S. Enger P. Ø. (2017). Drug repurposing in cancer. Pharmacol. Res. 124, 74–91. 10.1016/j.phrs.2017.07.013 28712971

[B82] Son D.-S. Lee E.-S. Adunyah S. E. (2020). The antitumor potentials of benzimidazole anthelmintics as repurposing drugs. Immune Netw. 20, e29. 10.4110/in.2020.20.e29 32895616 PMC7458798

[B83] Staedtke V. Gray-Bethke T. Riggins G. J. Bai R.-Y. (2020). Preventative effect of mebendazole against malignancies in neurofibromatosis 1. Genes (Basel) 11, 762. 10.3390/genes11070762 32650362 PMC7397152

[B84] Studenovský M. Rumlerová A. Kostka L. Etrych T. (2021). HPMA-based polymer conjugates for repurposed drug mebendazole and other imidazole-based therapeutics. Polym. (Basel) 13, 2530. 10.3390/polym13152530 PMC8347626 34372133

[B85] Sung S. J. Kim H.-K. Hong Y.-K. Joe Y. A. (2019). Autophagy is a potential target for enhancing the anti-angiogenic effect of mebendazole in endothelial cells. Biomol. Ther. Seoul. 27, 117–125. 10.4062/biomolther.2018.222 30642153 PMC6319545

[B86] Van Eerdenbrugh B. Stuyven B. Froyen L. Van Humbeeck J. Martens J. A. Augustijns P. (2009). Downscaling drug nanosuspension production: processing aspects and physicochemical characterization. AAPS PharmSciTech 10, 44–53. 10.1208/s12249-008-9170-5 19148764 PMC2663663

[B87] Ward R. A. Fawell S. Floc’h N. Flemington V. McKerrecher D. Smith P. D. (2021). Challenges and opportunities in cancer drug resistance. Chem. Rev. 121, 3297–3351. 10.1021/acs.chemrev.0c00383 32692162

[B88] Williamson T. Bai R.-Y. Staedtke V. Huso D. Riggins G. J. (2016). Mebendazole and a non-steroidal anti-inflammatory combine to reduce tumor initiation in a colon cancer preclinical model. Oncotarget 7, 68571–68584. 10.18632/oncotarget.11851 27612418 PMC5356574

[B89] Xiong J. Zhang T. Lan P. Zhang S. Fu L. (2022). Insight into the molecular mechanisms of gastric cancer stem cell in drug resistance of gastric cancer. Cancer Drug Resist 5, 794–813. 10.20517/cdr.2022.11 36176765 PMC9511795

[B90] Yauch R. L. Gould S. E. Scales S. J. Tang T. Tian H. Ahn C. P. (2008). A paracrine requirement for hedgehog signalling in cancer. Nature 455, 406–410. 10.1038/nature07275 18754008

[B91] Yaylim İ. Aru M. Farooqi A. A. Hakan M. T. Buttari B. Arese M. (2024). Regulation of Nrf2/Keap1 signaling pathway in cancer drug resistance by galectin-1: cellular and molecular implications. Cancer Drug Resist 7, 8. 10.20517/cdr.2023.79 38434765 PMC10905161

[B92] Zhang F. Li Y. Zhang H. Huang E. Gao L. Luo W. (2017). Anthelmintic mebendazole enhances cisplatin’s effect on suppressing cell proliferation and promotes differentiation of head and neck squamous cell carcinoma (HNSCC). Oncotarget 8, 12968–12982. 10.18632/oncotarget.14673 28099902 PMC5355070

[B93] Zhang L. Bochkur Dratver M. Yazal T. Dong K. Nguyen A. Yu G. (2019). Mebendazole potentiates radiation therapy in triple-negative breast cancer. Int. J. Radiat. Oncol. 103, 195–207. 10.1016/j.ijrobp.2018.08.046 PMC6457649 30196056

[B94] Zhang Z. Zhou L. Xie N. Nice E. C. Zhang T. Cui Y. (2020). Overcoming cancer therapeutic bottleneck by drug repurposing. Signal Transduct. Target. Ther. 5, 113. 10.1038/s41392-020-00213-8 32616710 PMC7331117

[B95] Zhou J. Giannakakou P. (2005). Targeting microtubules for cancer chemotherapy. Curr. Med. Chem. Agents 5, 65–71. 10.2174/1568011053352569 15720262

